# Characterization of a novel orthoreovirus isolated from fruit bat, China

**DOI:** 10.1186/s12866-014-0293-4

**Published:** 2014-11-30

**Authors:** Tingsong Hu, Wei Qiu, Biao He, Yan Zhang, Jing Yu, Xiu Liang, Wendong Zhang, Gang Chen, Yingguo Zhang, Yiyin Wang, Ying Zheng, Ziliang Feng, Yonghe Hu, Weiguo Zhou, Changchun Tu, Quanshui Fan, Fuqiang Zhang

**Affiliations:** Centre for Disease Control and Prevention, Chengdu Military Region, 168 Daguan Road, Kunming, 650032 China; Institute of Military Veterinary, Academy of Military Medical Sciences, Changchun, 130062 China; Department of Biochemistry and Molecular Biology, Fudan University Shanghai Medical College, Shanghai, 200030 China; The Animal Epidemic Disease Control Center, Kunming, 650032 Yunnan Province China; General Hospital of Chengdu Military Region of PLA, Chengdu, 610083 China

**Keywords:** Bat Orthoreovirus, Prevalence, Viral genome reassortment

## Abstract

**Background:**

In recent years novel human respiratory disease agents have been described for Southeast Asia and Australia. The causative pathogens were classified as pteropine orthoreoviruses with a strong phylogenetic relationship to orthoreoviruses of bat origin.

**Results:**

In this report, we isolated a novel Melaka-like reovirus (named “Cangyuan virus”) from intestinal content samples of one fruit bat residing in China’s Yunnan province. Phylogenetic analysis of the whole Cangyuan virus genome sequences of segments L, M and S demonstrated the genetic diversity of the Cangyuan virus. In contrast to the L and M segments, the phylogenetic trees for the S segments of Cangyuan virus demonstrated a greater degree of heterogeneity.

**Conclusions:**

Phylogenetic analysis indicated that the Cangyuan virus was a novel orthoreovirus and substantially different from currently known members of Pteropine orthoreovirus (PRV) species group.

**Electronic supplementary material:**

The online version of this article (doi:10.1186/s12866-014-0293-4) contains supplementary material, which is available to authorized users.

## Background

Many emerging infectious diseases are caused by zoonotic transmission, and the consequence is often unpredictable. Zoonoses have been well represented with the 2003 outbreak of severe acute respiratory syndrome (SARS) due to a novel coronavirus [1,2]. Bats are associated with an increasing number of emerging and reemerging viruses, many of which pose major threats to public health, in part because they are mammals which roost together in large populations and can fly over vast geographical distances [3,4]. Many distinct viruses have been isolated or detected (molecular) from bats including representatives from families *Rhabdoviridae*, *Paramyxoviridae*, *Coronaviridae*, *Togaviridae*, *Flaviviridae*, *Bunyaviridae*, *Reoviridae*, *Arenaviridae*, *Herpesviridae*, *Picornaviridae*, *Filoviridae, Hepadnaviridae* and *Orthomyxoviridae* [3-8].

The *Reoviridae* (respiratory enteric orphan viruses) comprise a large and diverse group of nonenveloped viruses containing a genome of segmented double-stranded RNA, and are taxonomically classified into 10 genera [9-13]. Orthoreoviruses are divided into two subgroups, fusogenic and nonfusogenic, depending on their ability to cause syncytium formation in cell culture, and have been isolated from a broad range of mammalian, avian, and reptilian hosts [10-14]. Members of the genus *Orthoreovirus* contain a genome with 10 segments of dsRNA; 3 large (L1-L3), 3 medium (M1-M3), and 4 small (S1 to S4) [15].

The discovery of Melaka and Kampar viruses, two novel fusogenic reoviruses of bat origin, marked the emergence of orthoreoviruses capable of causing acute respiratory disease in humans [9,16]. Subsequently, other related strains of bat-associated orthoreoviruses have also been reported, including Xi River virus from China [17,18]. Wong et al. isolated and characterized 3 fusogenic orthoreoviruses from three travelers who had returned from Indonesia to Hong Kong during 2007–2010 [19,20].

In the present study we isolated a novel reovirus from intestinal contents taken from one fruit bat ( *Rousettus leschenaultia*) in Yunnan province, China. In the absence of targeted sequencing protocols for a novel virus, we applied the VIDISCR (Virus-Discovery-cDNA RAPD) virus discovery strategy to confirm and identify a novel Melaka-like reovirus, the “Cangyuan virus”. To track virus evolution and to provide evidence of genetic reassortment PCR sequencing was conducted on each of the 10 genome segments, and phylogenetic analysis performed to determine genetic relatedness with other bat-borne fusogenic orthoreoviruses.

## Results

### Virus isolation and morphological characterization

**Figure 1:**
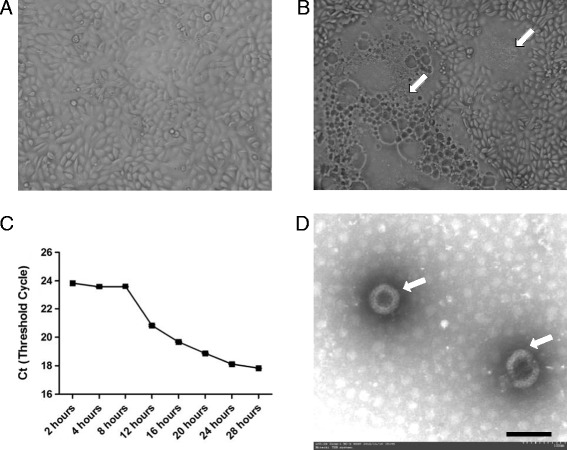
**Syncytium formation in Cangyuan virus-infected Vero E6 cells. (** **A** **)** Mock-infected. **(** **B** **)** Cangyuan virus –24 hours post-infection. **(** ***C*** **)** The viral growth curve of Cangyuan virus infected VeroE6 cells in the 28 hours. **(** **D** **)** Negatively stained electron micrograph of viral particles (arrowheads) recovered from the supernatant of Cangyuan virus-infected Vero E6 cells. Bar = 100 nm.

The Vero-E6 cells showed a syncytial cytopathic effect (CPE) after 24 hours of the first inoculation at 37°C (Figure 1). The virus was named “Cangyuan virus” after the location from which the host bats (*Rousettus leschenaultia*) were collected (Cangyuan city of Yunnan province, China). After the first passage in Vero E6 cells, Cangyuan virus began to cause syncytial CPE 24 hours post-inoculation; notably earlier than for Melaka virus and other orthoreovirus (Figure 1B) [9,16,17,20]. QPCR analysis demonstrated that the replication of Cangyuan virus began after 12 hours infected the Vero E6 cells (Figure 1C). After the second passage, Virus titrations were performed and the infectious dose of Cangyuan virus was 10^5.5^ TCID50/0.1 ml. QPCR analysis demonstrated that Cangyuan virus is the virus replicating in the cells and responsible for the observed CPE (Table 1).

**Table 1:** **QPCRs result of Cangyuan virus infected VeroE6 cells with the L2 segment primers**

***Cangyuan virus infected VeroE6 cells ( TCID50/0.1 mL)***	***Average of Ct (Threshold Cycle)***	***Time of CPE (hours)***
10^5.5^	16.667 ± 0.046	16
10^4.5^	17.957 ± 0.811	24
10^3.5^	20.273 ± 0.448	24
10^2.5^	22.817 ± 0.358	24

Note: The culture supernatants 0.1 mL (after the second passage,10^5.5^ TCID50/0.1 mL titer) was serially diluted until 10^−3^ and infected Vero E6 cells. After the 24 hours, the culture supernatants were analyzed by RT-QPCRs. The normal Vero-E6 cells as negative controls for RT-QPCRs.

Negative-staining EM of particles in the supernatant recovered from Vero E6 cells infected with Cangyuan virus revealed non-enveloped icosahedral virus-like particles, approximately 70–80 nm in diameter, possessing a double capsid with conspicuous “spikes” or “turrets” situated on the inner core; features characteristic of the family *Reoviridae*, genus *Orthoreovirus* (Figure 1D) [9].

### Neutralizing antibody titers

Serum samples from 50 fruit bats ( *Rousettus leschenaultia*) collected from Cangyuan city were screened for anti-Cangyuan virus neutralizing antibody. According to the Neutralizing Antibody Titers determined in this study, the serum of two bats had a neutralizing antibody titer of 1,280 against Cangyuan virus and the serum of five bats (10%, 5/50) a titer of 640 against Cangyuan virus. Our studies indicated a 26% (13/50) prevalence for antibody titers >1:160 for Cangyuan virus-specific antibodies in fruit bats (*Rousettus leschenaultia*). The control serum had a neutralizing antibody titer <10. At present, it is not clear whether Cangyuan virus is carried by a specific fruit bat species or by multiple bat species circulating in the region. A preliminary survey of bat sera collected in Cangyuan city of Yunnan province from our previous studies indicated a low prevalence of Cangyuan virus-specific antibodies in at least two different insectivorous bat species: *Rhinolophus luctus* (1/23, Antibody Titers = 1:320) and Small leaf-nosed bat (*Hipposideros pomona*) (1/30, Antibody Titers = 1:160).

### QPCR, nucleotide sequences and phylogeny

PCRs testing were repeated on the 50 fruit bats original samples including the Kidney, heart, lung, liver, spleen, intestine, rectal swab sample, and brain samples. Two bat’s QPCRs results were positive. One bat’s QPCRs result was positive in the lung, intestine sample (Cangyuan virus isolated) and rectal swab sample, and the Ct (Threshold Cycle) of QPCR were 19.86 ± 0.056, 19.52 ± 0.041, 19.64 ± 0.061 respectively. The Ct of another bat’s PCR were 23.07 ± 0.253, 22.53 ± 0.171 in the intestine sample and rectal swab sample, respectively.

**Figure 2:**
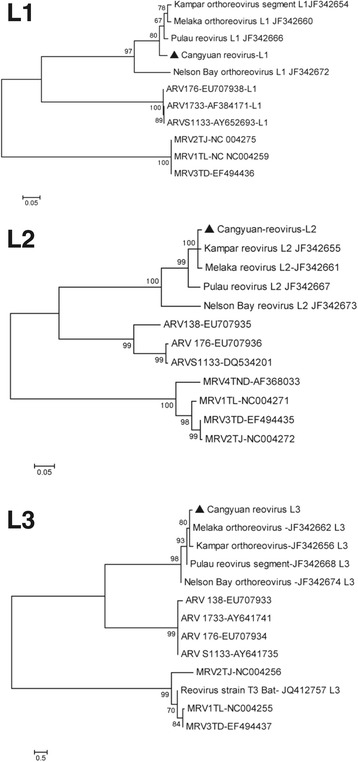
**Phylogenetic trees based on the nucleotide sequence of the L-class genome segments of orthoreoviruses.** GenBank accession numbers for each sequence are provided adjacent to the virus name. Numbers at nodes indicate levels of bootstrap support calculated from 1000 trees. The fragment length of the L1-L3 genome segments were 3885, 3820 and 3944 bp, respectively.

**Figure 3:**
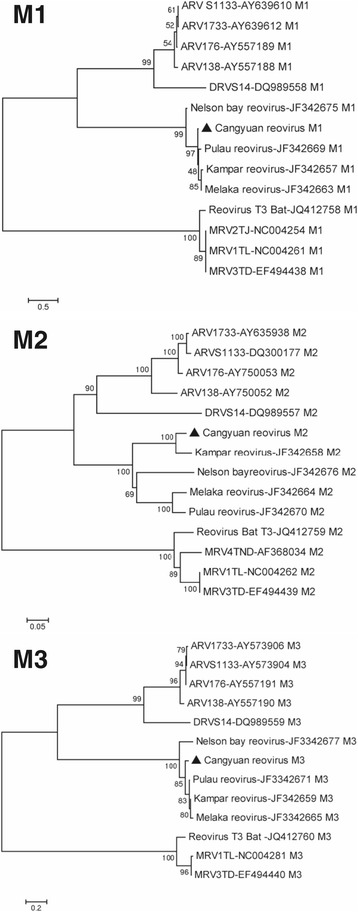
**Phylogenetic trees based on the nucleotide sequence of the M-class genome segments of orthoreoviruses.** GenBank accession numbers for each sequence are provided adjacent to the virus name. The fragment length of the M1-M3 genome segments were 2277, 2134 and 1983 bp, respectively. Numbers at nodes indicate levels of bootstrap support calculated from 1000 trees.

**Figure 4:**
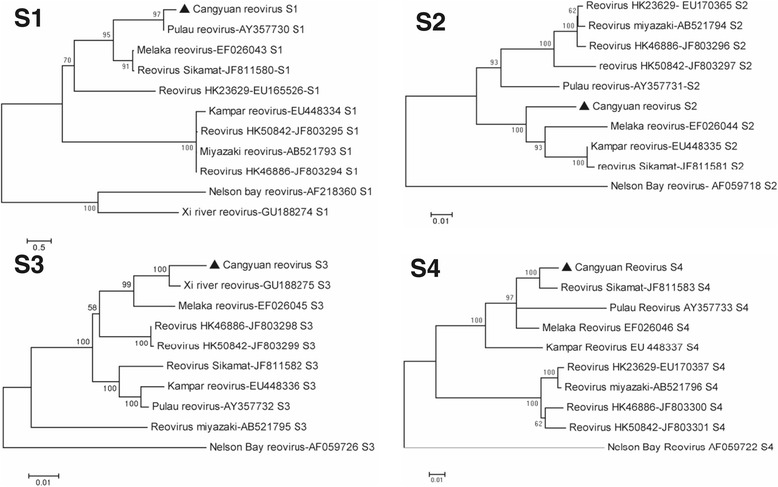
**Phylogenetic trees based on the nucleotide sequence of the S-class genome segments of orthoreoviruses**. GenBank accession numbers for each sequence are provided adjacent to the virus name. The fragment length of the S1-S4 genome segments were 1596, 1323,1180 and 1184 bp, respectively. Numbers at nodes indicate levels of bootstrap support calculated from 1000 trees.

To establish the evolutionary relationship between Cangyuan virus and other known orthoreoviruses, Homology were compared (Table 2, Table 3 and Additional file 1: Table S1, Additional file 2: Table S2 and Additional file 3: Table S3) and phylogenetic trees were constructed based on the nucleotide sequences of the L genome segments (Figure 2), the M genome segments (Figure 3) and the S genome segments (Figure 4). The Cangyuan virus L1-L3, M1-M3 segments sequence identity were *81.6%* –*94.2%*, *83.8%*–*97.9%*, *85.9%–97.6%* ( Additional file 1: Table S1), *82.2%–94.1%, 78. 1%–95.0%,* and *83.0%–93.9%* (Table 2, Additional file 2: Table 2), respectively, by alignment with Pteropine orthoreovirus (PRV) species group. The phylogenetic trees for L2, L3, M1 and M2 segments demonstrated that Cangyuan virus was most closely related to Melaka and Kampar viruses, and was placed in Pteropine orthoreovirus (PRV) species group which covers all known bat-borne orthoreoviruses together with Nelson Bay orthoreovirus [12,15,21].

**Table 2:** **Homology matrix of Cangyuan virus’s M2 gene segment with other fusogenic orthoreoviruses**

	***Homology matrix of Cangyuan virus’s M2 gene segment with other fusogenic orthoreoviruses***													
ARV138-AY750052_M2	100%													
ARV1733-AY635938_M2	86.6%	100%												
ARV176-AY750053_M2	90.0%	95.7%	100%											
ARVS1133-DQ300177_M2	86.4%	98.6%	95.3%	100%										
*Cangyuan-KC994907_M2*	*64.3%*	*64.0%*	*63.4%*	*63.7%*	*100%*									
DRVS14-DQ989557_M2	68.9%	68.7%	68.6%	68.8%	*61.3%*	100%								
Kampar-JF342658_M2	64.1%	63.4%	63.2%	63.2%	*95.0%*	61.5%	100%							
Melaka-JF342664_M2	63.8%	62.6%	62.4%	62.3%	*79.2%*	61.3%	78.9%	100%						
MRV1TL-NC004262_M2	50.4%	50.4%	50.4%	50.2%	*49.9%*	49.3%	49.4%	49.2%	100%					
MRV3TD-EF494439_M2	50.5%	50.4%	50.4%	50.3%	*49.7%*	49.2%	49.3%	49.0%	99.7%	100%				
MRV4TND-AF368034_M2	51.3%	50.6%	51.3%	50.6%	*49.3%*	49.1%	49.3%	49.8%	91.2%	91.5%	100%			
Nelson_bay-JF342676_M2	63.5%	62.9%	62.9%	62.9%	*78.1%*	61.0%	78.8%	79.7%	48.9%	48.7%	48.8%	100%		
Pulau-JF342670_M2	63.0%	62.5%	62.1%	62.3%	*79.2%*	61.8%	78.8%	94.2%	49.7%	49.6%	49.9%	79.7%	100%	
Bat_T3-JQ412759_M2	51.3%	50.9%	51.1%	50.8%	*49.2%*	50.1%	49.0%	49.8%	90.8%	91.1%	89.4%	49.0%	50.1%	100%

**Table 3:** **Homology comparison of Cangyuan virus’s S1 gene segments nucleotide sequences with other fusogenic orthoreovirus**

	***Homology matrix of Cangyuan virus’s S1 gene segments with other fusogenic orthoreovirus***										
Cangyuan -KC994909_ S1	100%										
Kampar _EU448334_S1	66.2%	100%									
Melaka _EF026043_S1	79.9%	64.5%	100%								
miyazaki- AB521793_S1	65.4%	94.5%	64.5%	100%							
Nelson_Bay _AF218360_S1	55.3%	55.1%	54.9%	54.2%	100%						
Pulau _ AY357730_S1	94.7%	66.9%	79.4%	66.1%	55.3%	100%					
HK23629- EU165526-S1	70.5%	64.2%	70.0%	65.0%	53.3%	69.8%	100%				
HK46886- JF803294_S1	64.4%	94.5%	63.6%	99.8%	53.5%	65.1%	65.0%	100%			
HK50842- JF803295_S1	64.5%	94.3%	63.8%	99.4%	53.6%	65.1%	65.0%	99.6%	100%		
Sikamat- JF811580-S1	80.1%	64.1%	95.9%	64.3%	55.5%	79.4%	70.2%	63.4%	63.5%	100%	
Xi_river-GU188274_S1	56.1%	55.0%	56.1%	54.4%	65.4%	54.8%	53.8%	53.4%	53.6%	55.9%	100%

To better understand the genetic relatedness of Cangyuan virus to other known bat-borne orthoreoviruses, the published sequences for the S genome segment of bat-borne orthoreoviruses known for causing acute respiratory disease in humans were retrieved from GenBank and used to compare homology (Table 3 and Additional file 2: Table S2) and construct phylogenetic trees (Figure 4). The Cangyuan virus S1-S4 segments sequence identity were 55.3%–94.7%, 86.2%–95.5%, 86.5%–97.9%%, and 83.5%–98.2%, respectively (Table 3 and Additional file 2: Table S2). The S1 segment demonstrated a greater heterogeneity than other S segments in Pteropine orthoreovirus (PRV) species group.

## Discussion

The discovery of Melaka and Kampar viruses provide evidence that a novel group of fusogenic orthoreoviruses of bat origin are associated with acute respiratory disease in humans [9,16]. To date, there have been six confirmed outbreaks of human respiratory illness caused by this group of viruses; three in Malaysia and three in Bali/Hong Kong [9,16,17,19,20]. Despite a lack of direct epidemiological and clinical evidence to support the novel Cangyuan orthoreovirus of bat origin isolated in this study as a causative agent of human respiratory tract infections in China, the genetic relatedness between Cangyuan virus and the Melaka and Kampar viruses implies that it may be a zoonotic infectious agent of clinical significance to human health.

One possibility for potential virulence in humans is genome segment reassortment, which is common among segmented RNA viruses, including the reoviruses [9,15,20]. Reassortment has been reported for avian and mammalian reoviruses [22-25]. Phylogenetic analysis of the full sequences of segments L, M and S demonstrated the genetic diversity of the Cangyuan virus.

As summarized above and in Figure 2, 3 and 4, the phylogenetic trees derived from the S segments demonstrated a greater heterogeneity than those derived for the corresponding L and M segments (Table 2, Table 3 and Additional file 2: Table S1, Additional file 2: Table S2 and Additional file 3: Table S3). All four possible topologies were observed for the S segments of the ten strains: Cangyuan virus was clustered with the Pulau virus (S1: the S1 virus cell attachment protein); Cangyuan virus and Melaka virus clustered together (S2: the S2 major inner capsid protein); Cangyuan virus and Xi river virus together (S3: the S3 nonstructural protein); and Cangyuan virus clustered with Sikamat virus (S4: the S4 Major component of outer capsid).

The distinctive topology pattern of the phylogenetic tree based on the M2 segments (Major outer-capsid protein) is especially interesting and may indicate two important findings [9,15-17]. First, it further confirms the notion, derived from the topology of the S segment trees, that genetic reassortment may have occurred among the Malaka related orthoreoviruses [15,22-25]. Second, the Cangyuan virus M2 segment shared sequence identities of 95.0, 79.2, 79.2, and 78. 1% with the M2 segments of Kampar, Malaka, Pulau and Nelson Bay viruses, respectively (Table 2). The tree based on M2 segments indicates a great divergence of the Cangyuan and Kampar virus segments from those of the other three strains. This may suggest a new genotype represented by the Cangyuan and Kampar virus M2 segment [15]. The difference in sequence homology between segments of the reovirus strains suggested that genetic reassortment may have occurred among the bat-borne Melaka related orthoreoviruses.

## Conclusion

In conclusion, phylogenetic analysis demonstrated that the Cangyuan virus was closely related to the Malaka and Kampar viruses and that a prevalence of Cangyuan virus-specific antibodies was present in fruit bat families circulating in Yunnan province, China. This further highlights the urgent need to systematically survey bat-borne viruses in south China so as to enable us to conduct more effective risk assessment, to provide forecast and devise better prevention and control strategies for potential future outbreaks.

## Methods

### Sample, serum and organ collection

Fifty fruit bats (***Rousettus leschenaultia***) were collected from across eight different locations in Cangyuan city of China’s Yunnan province. All of the locations including three lychee, two mango orchards and three caverns were in close proximity to human residences. All bats were euthanized by intracardiac injection of sodium pentobarbital. Following euthanasia, serum samples were collected by cardiac puncture. The serum samples were used for neutralizing-antibody tests. Necropsies were performed directly following euthanasia. The necropsy findings demonstrated that these bats were apparently healthy and no special pathological lesions was observed (data not show). The intestine samples were used for virus isolation and the kidney, heart, lung, liver, spleen, rectal swab and brain samples were processed for PCRs testing. The tissue samples stored at -80°C.

### Ethics statement

All procedures using animals were approved by the Animal Care and Use Committees of Centre for Disease Control and Prevention, Chengdu Military Region and were in compliance with the China Animal Welfare Act. We state clearly that no specific permissions were required for these locations/activities and confirm that the field studies did not involve endangered or protected species.

### Virological investigation

Virus isolation was carried out in Vero E6 cells. To refer [23], kohl et al [26], Vero E6 cells were maintained at 37°C with 5% CO_2_ unless stated otherwise. Cells were seeded into 24-well cell-culture plates and incubated overnight until 80–90% confluent. For infection, homogenates of the intestine samples for each bat were centrifuged at low-speed (6,000 × g) for 10 min at 4°C, treated with 100,000 U/ml penicillin and 100 μg/ml streptomycin, and then inoculated into Vero E6 cells for 1 hour under standard cell-culture conditions and washed the cells three times with PBS (phosphate buffer saline) after inoculation. The infected cells were sub-cultured three times and observed daily for the occurrence of cytopathic effects (CPE). Upon CPE in the third subcultivation, the supernatant was passaged in 80–90% confluent fresh Vero cells in a 175 cm^2^ flask for one week. Aliquots were stored at -80°C. One aliquot was titrated on Vero E6 cells to estimate the viral titer. After the second passage, Virus titrations were performed by end-point titration in VeroE6 cells and the TCID50/0.1 ml was calculated from 5 replicates by the method of Spearman-Karber.

### Identification of virus by electron microscopy (EM)

Cangyuan virus was observed by EM. For negative staining, paraformaldehyde-fixed (2%) purified Cangyuan virus was adsorbed onto carbon-coated parlodion-filmed nickel grids and negatively stained with 2% phosphotungstic acid. Specimens were examined using a transmission electron microscope (Hitachi-8100, Japan) at 80 kV.

### VIDISCR, PCR, nucleotide sequencing, and phylogenetic analysis

Culture supernatants of Cangyuan virus was analyzed by VIDISCR. Virus particles were harvested from cells by three freeze-thaw cycles and the resulting suspension purified from cell debris by low-speed centrifugation. Nucleic acids were extracted using the AxyPrep Body Fluid Viral DNA/RNA Miniprep Kit (Axygen, Inc.). Reverse transcription of the viral RNA was performed by using the RevertAid™ First Strand cDNASynthesis Kit (Fermentas, Inc). The VIDISCR assay was performed as previously described [27]. To further characterize the virus and its phylogeny primers were designed with Primer Premier 5.0 based on published sequences selective for the 10 genome segments of Melaka virus and other orthoreoviruses (Additional file 4: Table S4) [9,16]. Each of the 10 genome segments were amplified using the pfu PCR Polymerase Kit (Fermentas, Inc) with the primers as listed in Additional file 4: Table S4. Reverse transcription PCR was performed as described [9,16]. The amplicons were visualized using 2% agarose gel electrophoresis. PCR products were sequenced after cloning by using CloneJET™ PCR cloning kit (Fermentas, Inc). Sequence alignment was conducted using DNAMAN5.0 and phylogenetic analysis of the whole Cangyuan virus genome sequences of all L, M and S segments were performed by the neighbor-joining method using MEGA6 software (www.megasoftware.net). The Phylogenetic data have been deposited in TreeBase (Study Accession URL: http://purl.org/phylo/treebase/phylows/study/TB2:S16635). RT-QPCRs testing were repeated on the 50 fruit bats original samples including the Kidney, heart, lung, liver, spleen, intestine, rectal swab sample, and brain samples with the L2 segment primers (CY-L2QF1: 5′ GCA ATG CCG AAT ATC TAA AGC 3′, CY-L2QR1:5′ AGA GCA AGA GCC CAA ATG AA 3′). The reaction was performed using the One Step SYBR® PrimeScript™ PLUS RT-PCR Kit (TAKARA BIOTECHNOLOGY (Dalian) CO., LTD) by lightcycler2.0 (Roche). To test the viral growth curve of the Cangyuan virus, the virus samples were harvested at 2, 4, 8, 12, 16, 20, 24 and 28 hours after infection and quantified with the already established RT-QPCR. To test whether the Cangyuan virus is the virus (or one of the viruses) replicating in the cells and responsible for the observed CPE, the culture supernatants 0.1 mL (after the second passage,10^5.5^ TCID50/0.1 mL titer) was serially diluted until 10^−3^ and infected Vero E6 cells. After the 24 hours, the culture supernatants were analyzed by RT-QPCRs with the L2 segment primers.

### Virus neutralization test

To refer the Chua’s report [9], Serum samples from each bat were screened for anti-Cangyuan virus neutralizing antibody. Serum, the negative control (fetal calf serum, FCS), virus, and cell controls were included in the assay. Serial 2-fold dilutions (1:10 to 1:640) of control and fruit bat sera were prepared in duplicate. An equal volume of Cangyuan virus working stock containing 150 TCID50 was added to the diluted sera and incubated for 1 hour. The preincubated virus/serum mix was added to 80–90% confluent Vero cell monolayers. After 1 hour of virus adsorption, the inoculum was removed, the monolayers washed three times with 2%FCS MEM, and fresh media added. All incubations were performed under standard cell culture conditions. Vero cell monolayers were observed for CPE 3 days post-inoculation. The ability of sera to neutralize virus was determined by scoring the extent of CPE observed for duplicate wells.

## Additional files

Additional file 1: Table S1. Homology comparison of Cangyuan virus’s L gene segments nucleotide sequences with other fusogenic orthoreovirus. Additional file 2: Table S2. Homology comparison of Cangyuan virus’s M gene segments nucleotide sequences with other fusogenic orthoreovirus. Additional file 3: Table S3. Homology comparison of Cangyuan virus’s S gene segments nucleotide sequences with other fusogenic orthoreovirus. Additional file 4: Table S4. Primers used to amplify the 10 gene segments of fusogenic orthoreovirus and the GenBank accession numbers of Orthoreovirus strain Cangyuan.
