# Supplementary material for: Characterization of a novel orthoreovirus isolated from fruit bat, China
Source: BMC Microbiol. 2014 Nov 30;14:293. doi: 10.1186/s12866-014-0293-4 (PMC4264558; doi:10.1186/s12866-014-0293-4)
Supplement: Additional file 1: Table S1. — Homology comparison of Cangyuan virus’s L gene segments nucleotide sequences with other fusogenic orthoreovirus. [file 12866_2014_293_MOESM1_ESM.docx]

**Additional file 1: Table S1. Homology comparison of Cangyuan virus’s L gene segments nucleotide sequences with other fusogenic orthoreovirus**

|  | **Homology matrix of Cangyuan virus’s L1 gene segments with other fusogenic orthoreovirus** | | | | | | | | | | | |
| --- | --- | --- | --- | --- | --- | --- | --- | --- | --- | --- | --- | --- |
| ARV1733-AF384171-L1 | 100% |  |  |  |  |  |  |  |  |  |  |  |
| ARV176-EU707938-L1 | 99.6% | 100% |  |  |  |  |  |  |  |  |  |  |
| ARVS1133-AY652693-L1 | 99.8% | 99.6% | 100% |  |  |  |  |  |  |  |  |  |
| ***Cangyuan-KC994903-L1*** | ***48.2%*** | ***48.3%*** | ***48.1%*** | ***100%*** |  |  |  |  |  |  |  |  |
| Kampar-JF342654_L1 | 47.8% | 47.9% | 47.8% | ***93.1%*** | 100% |  |  |  |  |  |  |  |
| Melaka-JF342660_L1 | 47.5% | 47.6% | 47.6% | ***93.5%*** | 98.0% | 100% |  |  |  |  |  |  |
| MRV1TL-NC_NC004259 | 40.5% | 40.6% | 40.7% | ***42.6%*** | 41.8% | 41.9% | 100% |  |  |  |  |  |
| MRV2TJ-NC_004275 | 40.5% | 40.6% | 40.7% | ***42.6%*** | 41.8% | 41.9% | 100.0% | 100% |  |  |  |  |
| MRV3TD-EF494436 | 40.5% | 40.6% | 40.7% | ***42.7%*** | 41.9% | 42.0% | 99.8% | 99.8% | 100% |  |  |  |
| Nelson_bay- JF342672_L1 | 47.4% | 47.4% | 47.4% | ***81.6%*** | 82.1% | 81.9% | 42.1% | 42.1% | 42.1% | 100% |  |  |
| Pulau-JF342666_L1 | 47.8% | 47.9% | 47.9% | ***94.2%*** | 97.3% | 97.9% | 42.1% | 42.1% | 42.2% | 81.9% | 100% |  |
| T3_Bat-JQ412755_L1 | 31.9% | 32.0% | 32.0% | ***33.7%*** | 33.4% | 33.2% | 31.6% | 31.6% | 31.5% | 32.5% | 33.4% | 100% |

|  | **Homology matrix of Cangyuan virus’s L2 gene segments with other fusogenic orthoreovirus** | | | | | | | | | | | | |
| --- | --- | --- | --- | --- | --- | --- | --- | --- | --- | --- | --- | --- | --- |
| ARV_176-EU707936 | 100% |  |  |  |  |  |  |  |  |  |  |  |  |
| ARV138-EU707935 | 85.0% | 100% |  |  |  |  |  |  |  |  |  |  |  |
| ARVS1133-DQ534201 | 99.3% | 84.7% | 100% |  |  |  |  |  |  |  |  |  |  |
| ***Cangyuan-L2-KC994904*** | ***64.6%*** | ***64.6%*** | ***63.6%*** | ***100%*** |  |  |  |  |  |  |  |  |  |
| Kampar-L2_JF342655 | 63.3% | 64.6% | 63.3% | ***97.9%*** | 100% |  |  |  |  |  |  |  |  |
| Melaka-L2-JF342661 | 63.3% | 64.5% | 63.3% | ***97.9%*** | 98.6% | 100% |  |  |  |  |  |  |  |
| MRV1TL-NC004271 | 54.4% | 54.3% | 54.2% | ***53.9%*** | 53.7% | 53.7% | 100% |  |  |  |  |  |  |
| MRV2TJ-NC004272 | 54.3% | 54.2% | 54.1% | ***53.8%*** | 53.6% | 53.5% | 96.0% | 100% |  |  |  |  |  |
| MRV3TD-EF494435 | 54.3% | 54.2% | 54.1% | ***53.9%*** | 53.7% | 53.6% | 96.1% | 99.7% | 100% |  |  |  |  |
| MRV4TND-AF368033 | 54.7% | 54.6% | 54.6% | ***54.0%*** | 53.6% | 53.6% | 89.2% | 89.2% | 89.5% | 100% |  |  |  |
| Nelson_bay- L2_JF342673 | 63.3% | 63.7% | 63.4% | ***83.8%*** | 83.6% | 83.8% | 53.0% | 53.0% | 53.0% | 52.7% | 100% |  |  |
| Pulau-L2_JF342673 | 63.1% | 64.4% | 63.1% | ***94.0%*** | 94.6% | 94.6% | 53.9% | 53.7% | 53.8% | 53.4% | 83.5% | 100% |  |
| T3_Bat-JQ412756_L2 | 35.5% | 35.1% | 35.3% | ***36.2%*** | 35.4% | 35.5% | 34.6% | 34.5% | 34.5% | 34.0% | 35.3% | 35.3% | 100% |

|  | **Homology matrix of Cangyuan virus’s L3 gene segments with other fusogenic orthoreovirus** | | | | | | | | | | | | |
| --- | --- | --- | --- | --- | --- | --- | --- | --- | --- | --- | --- | --- | --- |
| ARV_138-EU707933 | 100% |  |  |  |  |  |  |  |  |  |  |  |  |
| ARV_1733-AY641741 | 91.4% | 100% |  |  |  |  |  |  |  |  |  |  |  |
| ARV_176-EU707934 | 91.4% | 99.7% | 100% |  |  |  |  |  |  |  |  |  |  |
| ARV_S1133-AY641735 | 91.4% | 100.0% | 99.7% | 100% |  |  |  |  |  |  |  |  |  |
| ***Cangyuan-L3-KC994905*** | ***61.0%*** | ***61.3%*** | ***61.2%*** | ***61.2%*** | ***100%*** |  |  |  |  |  |  |  |  |
| Kampar-L3-JF342656 | 66.7% | 66.6% | 66.5% | 66.6% | ***94.7%*** | 100% |  |  |  |  |  |  |  |
| Melaka-L3-JF342662 | 66.8% | 67.0% | 67.0% | 67.0% | ***97.6%*** | 95.3% | 100% |  |  |  |  |  |  |
| MRV1TL-NC004255 | 48.7% | 49.5% | 49.4% | 49.4% | ***48.4%*** | 48.5% | 48.8% | 100% |  |  |  |  |  |
| MRV2TJ-NC004256 | 49.3% | 49.9% | 49.8% | 49.8% | ***48.1%*** | 48.6% | 49.0% | 76.5% | 100% |  |  |  |  |
| MRV3TD-EF494437 | 48.8% | 49.4% | 49.3% | 49.3% | ***48.9%*** | 48.6% | 48.9% | 97.7% | 76.5% | 100% |  |  |  |
| Nelson_bay- L3_JF342674 | 66.3% | 66.8% | 66.7% | 66.7% | ***85.9%*** | 85.5% | 86.1% | 49.7% | 49.7% | 49.5% | 100% |  |  |
| Pulau-L3_JF342668 | 66.4% | 66.5% | 66.5% | 66.5% | ***93.0%*** | 93.1% | 93.6% | 49.3% | 49.5% | 49.3% | 86.0% | 100% |  |
| T3_Bat-_JQ412757_L3 | 49.1% | 49.5% | 49.4% | 49.4% | ***48.3%*** | 48.5% | 48.9% | 87.6% | 77.4% | 87.6% | 49.4% | 49.3% | 100% |
