# Supplementary material for: Characterization of a novel orthoreovirus isolated from fruit bat, China
Source: BMC Microbiol. 2014 Nov 30;14:293. doi: 10.1186/s12866-014-0293-4 (PMC4264558; doi:10.1186/s12866-014-0293-4)
Supplement: Additional file 2: Table S2. — Homology comparison of Cangyuan virus’s M gene segments nucleotide sequences with other fusogenic orthoreovirus. [file 12866_2014_293_MOESM2_ESM.docx]

**Additional file 2: Table S2. Homology comparison of Cangyuan virus’s M gene segments nucleotide sequences with other fusogenic orthoreovirus**

|  | **Homology matrix of Cangyuan virus’s M1 gene segment with other fusogenic orthoreoviruses** | | | | | | | | | | | | | |
| --- | --- | --- | --- | --- | --- | --- | --- | --- | --- | --- | --- | --- | --- | --- |
| S1133-AY639610_M1 | 100% |  |  |  |  |  |  |  |  |  |  |  |  |  |
| ARV138-AY557188_M1 | 90.4% | 100% |  |  |  |  |  |  |  |  |  |  |  |  |
| ARV1733-AY639612_M1 | 99.9% | 90.0% | 100% |  |  |  |  |  |  |  |  |  |  |  |
| ARV176-AY557189_M1 | 98.4% | 91.8% | 98.5% | 100% |  |  |  |  |  |  |  |  |  |  |
| ***Cangyuan-KC994906_M1*** | ***52.9%*** | ***52.6%*** | ***52.6%*** | ***53.0%*** | ***100%*** |  |  |  |  |  |  |  |  |  |
| DRVS14-DQ989558_M1 | 73.8% | 73.9% | 73.1% | 73.5% | ***53.1%*** | 100% |  |  |  |  |  |  |  |  |
| Kampar-JF342657_M1 | 52.0% | 51.6% | 51.7% | 52.3% | ***94.1%*** | 52.5% | 100% |  |  |  |  |  |  |  |
| Melaka-JF342663_M1 | 52.1% | 51.8% | 51.8% | 52.4% | ***93.9%*** | 52.5% | 98.8% | 100% |  |  |  |  |  |  |
| MRV1TL-NC004261_M1 | 43.7% | 43.8% | 43.8% | 43.9% | ***43.5%*** | 43.1% | 43.9% | 43.7% | 100% |  |  |  |  |  |
| MRV2TJ-NC004254_M1 | 43.7% | 43.8% | 43.8% | 43.9% | ***43.5%*** | 43.1% | 43.9% | 43.7% | 100.0% | 100% |  |  |  |  |
| MRV2TJ-NC004254_M1 | 43.7% | 43.7% | 43.8% | 43.9% | ***43.4%*** | 43.0% | 43.8% | 43.7% | 99.7% | 99.7% | 100% |  |  |  |
| Nelson_bay-JF342675_M1 | 52.2% | 51.6% | 51.9% | 52.2% | ***82.2%*** | 51.9% | 82.9% | 82.7% | 43.2% | 43.2% | 43.2% | 100% |  |  |
| Pulau-JF342669_M1 | 52.9% | 51.8% | 52.7% | 53.0% | ***91.5%*** | 52.3% | 92.6% | 92.4% | 44.0% | 44.0% | 43.9% | 82.9% | 100% |  |
| T3_Bat-JQ412758_M1 | 43.7% | 43.5% | 43.8% | 43.7% | ***43.2%*** | 42.8% | 44.0% | 43.5% | 87.8% | 87.8% | 87.8% | 43.1% | 44.3% | 100% |

|  | **Homology matrix of Cangyuan virus’s M3 gene segment with other fusogenic orthoreoviruses** | | | | | | | | | | | | |
| --- | --- | --- | --- | --- | --- | --- | --- | --- | --- | --- | --- | --- | --- |
| ARV138-AY557190_M3 | 100% |  |  |  |  |  |  |  |  |  |  |  |  |
| ARV1733-AY573906_M3 | 92.3% | 100% |  |  |  |  |  |  |  |  |  |  |  |
| ARV176-AY557191_M3 | 93.6% | 98.2% | 100% |  |  |  |  |  |  |  |  |  |  |
| ARVS1133-AY573904_M3 | 92.6% | 99.5% | 98.6% | 100% |  |  |  |  |  |  |  |  |  |
| ***Cangyuan-KC994908_M3*** | ***48.8%*** | ***48.4%*** | ***48.5%*** | ***48.4%*** | ***100%*** |  |  |  |  |  |  |  |  |
| DRVS14-DQ989559_M3 | 71.7% | 72.1% | 72.1% | 72.4% | ***47.2%*** | 100% |  |  |  |  |  |  |  |
| *Kampar-JF342659_M3* | 48.7% | 47.9% | 48.3% | 47.9% | ***93.8%*** | 46.8% | 100% |  |  |  |  |  |  |
| Melaka-JF3342665_M3 | 48.3% | 47.6% | 48.0% | 47.5% | ***93.5%*** | 46.9% | 98.8% | 100% |  |  |  |  |  |
| MRV1TL-NC004281_M3 | 41.3% | 40.7% | 40.8% | 40.8% | ***36.3%*** | 40.9% | 36.1% | 36.1% | 100% |  |  |  |  |
| MRV3TD-EF494440_M3 | 41.2% | 40.5% | 40.7% | 40.7% | ***36.6%*** | 40.8% | 36.2% | 36.2% | 99.6% | 100% |  |  |  |
| Nelson_bay-JF3342677_M3 | 48.7% | 47.9% | 48.0% | 47.9% | ***83.0%*** | 47.0% | 82.8% | 83.1% | 36.7% | 36.7% | 100% |  |  |
| Pulau-JF3342671_M3 | 48.6% | 47.9% | 48.3% | 47.9% | ***93.9%*** | 46.5% | 96.2% | 96.0% | 36.2% | 36.3% | 83.2% | 100% |  |
| T3_Bat_-JQ412760_M3 | 41.5% | 40.6% | 40.9% | 40.8% | ***38.7%*** | 40.6% | 38.1% | 37.7% | 85.1% | 85.2% | 37.6% | 38.1% | 100% |
