# Supplementary material for: Characterization of a novel orthoreovirus isolated from fruit bat, China
Source: BMC Microbiol. 2014 Nov 30;14:293. doi: 10.1186/s12866-014-0293-4 (PMC4264558; doi:10.1186/s12866-014-0293-4)
Supplement: Additional file 3: Table S3. — Homology comparison of Cangyuan virus’s S gene segments nucleotide sequences with other fusogenic orthoreovirus. [file 12866_2014_293_MOESM3_ESM.docx]

**Additional file 3: Table S3. Homology comparison of Cangyuan virus’s S gene segments nucleotide sequences with other fusogenic orthoreovirus**

|  | **Homology matrix of Cangyuan virus’s S2 gene segments with other fusogenic orthoreovirus** | | | | | | | | | |
| --- | --- | --- | --- | --- | --- | --- | --- | --- | --- | --- |
| Cangyuan _S2-KC994910 | **100%** |  |  |  |  |  |  |  |  |  |
| Kampar _EU448335_S2 | **95.5%** | 100% |  |  |  |  |  |  |  |  |
| Melaka _EF026044_S2 | **94.4%** | 95.5% | 100% |  |  |  |  |  |  |  |
| Nelson_Bay _AF059718_S2 | **86.2%** | 86.1% | 85.9% | 100% |  |  |  |  |  |  |
| Pulau _AY357731-S2 | **91.8%** | 92.1% | 92.2% | 86.9% | 100% |  |  |  |  |  |
| HK23629-EU170365_S2 | **91.1%** | 90.4% | 90.4% | 85.8% | 93.1% | 100% |  |  |  |  |
| HK46886-JF803296_S2 | **90.8%** | 90.3% | 90.1% | 85.9% | 93.0% | 99.4% | 100% |  |  |  |
| HK50842-JF803297_S2 | **90.4%** | 89.8% | 89.6% | 86.4% | 92.5% | 96.9% | 96.9% | 100% |  |  |
| miyazaki-AB521794_S2 | **91.3%** | 90.6% | 90.7% | 86.4% | 93.4% | 99.5% | 99.4% | 96.9% | 100% |  |
| Sikamat-JF811581_S2 | **95.2%** | 99.5% | 95.2% | 86.0% | 92.0% | 90.1% | 90.0% | 89.5% | 90.3% | 100% |

|  | **Homology matrix of Cangyuan virus’s S3 gene segments with other fusogenic orthoreovirus** | | | | | | | | | | |
| --- | --- | --- | --- | --- | --- | --- | --- | --- | --- | --- | --- |
| Cangyuan -KC994911_S3 | **100%** |  |  |  |  |  |  |  |  |  |  |
| Kampar _EU448336_S3 | **92.6%** | 100% |  |  |  |  |  |  |  |  |  |
| Melaka _EF026045_S3 | **95.3%** | 94.4% | 100% |  |  |  |  |  |  |  |  |
| Nelson_Bay _AF059726_S3 | **86.5%** | 87.7% | 86.8% | 100% |  |  |  |  |  |  |  |
| Pulau _AY357732_S3 | **93.1%** | 98.7% | 94.5% | 88.1% | 100% |  |  |  |  |  |  |
| HK23629-EU170366_S3 | **88.6%** | 89.4% | 89.2% | 86.8% | 89.9% | 100% |  |  |  |  |  |
| HK46886-JF803298_S3 | **93.2%** | 94.5% | 94.2% | 86.7% | 95.1% | 90.1% | 100% |  |  |  |  |
| HK50842-JF803299_S3 | **93.1%** | 94.4% | 94.2% | 86.6% | 95.0% | 90.0% | 99.9% | 100% |  |  |  |
| miyazaki-AB521795_S3 | **89.3%** | 89.8% | 89.9% | 87.0% | 90.3% | 99.7% | 90.3% | 90.2% | 100% |  |  |
| Sikamat-JF811582_S3 | **92.8%** | 96.3% | 94.0% | 87.5% | 96.9% | 89.5% | 94.2% | 94.1% | 90.1% | 100% |  |
| Xiriver-GU188275_S3 | **97.9%** | 93.5% | 96.2% | 87.2% | 94.0% | 89.8% | 94.1% | 94.0% | 90.3% | 93.3% | 100% |

|  | **Homology matrix of Cangyuan virus’s S4 gene segments with other fusogenic orthoreovirus** | | | | | | | | | |
| --- | --- | --- | --- | --- | --- | --- | --- | --- | --- | --- |
| Cangyuan _S4-KC994912 | **100%** |  |  |  |  |  |  |  |  |  |
| Kampar _EU_448337_S4 | **92.7%** | 100% |  |  |  |  |  |  |  |  |
| Melaka _EF026046_S4 | **96.3%** | 95.8% | 100% |  |  |  |  |  |  |  |
| Nelson_Bay _AF059722_S4 | **83.5%** | 83.4% | 84.0% | 100% |  |  |  |  |  |  |
| Pulau _AY357733_S4 | **92.9%** | 91.1% | 94.3% | 82.4% | 100% |  |  |  |  |  |
| HK23629-EU170367 | **87.8%** | 88.0% | 88.0% | 82.7% | 85.5% | 100% |  |  |  |  |
| HK46886-JF803300_S4 | **87.5%** | 88.1% | 88.1% | 82.5% | 85.8% | 97.5% | 100% |  |  |  |
| HK50842-JF803301_S4 | **87.4%** | 87.9% | 87.6% | 82.7% | 85.4% | 97.8% | 97.9% | 100% |  |  |
| miyazaki-AB521796_S4 | **87.7%** | 88.0% | 87.9% | 82.7% | 85.0% | 99.5% | 97.5% | 97.6% | 100% |  |
| Sikamat-JF811583_S4 | **98.2%** | 92.8% | 96.2% | 83.6% | 93.0% | 87.5% | 87.1% | 87.0% | 87.5% | 100% |
