# Supplementary material for: Characterization of a novel orthoreovirus isolated from fruit bat, China
Source: BMC Microbiol. 2014 Nov 30;14:293. doi: 10.1186/s12866-014-0293-4 (PMC4264558; doi:10.1186/s12866-014-0293-4)
Supplement: Additional file 4: Table S4. — Primers used to amplify the 10 gene segments of fusogenic orthoreovirus and the GenBank accession numbers of Orthoreovirus strain Cangyuan. [file 12866_2014_293_MOESM4_ESM.docx]

**Additional file 4: Table S4: Primers used to amplify the 10 gene segments of fusogenic orthoreovirus and the GenBank accession numbers of Orthoreovirus strain Cangyuan**

| ***Primers name*** | ***Sequence*** | ***Length of* gene segment *(bp)*** | ***GenBank accession numbers*** |
| --- | --- | --- | --- |
| CY-REOV_L1_1324F | CGCTGTCAGTGATCGTCATG | L1: 3885 | KM382259 |
| CY-REOV_L1_1468R | CGCAAGTCAGATTGGCAGTA |  |  |
| CY-REOV_L1_1F | GCTTTATCACTCATGGCTCAGATT |  |  |
| CY-REOV_L1_2288F | GATTCGAGGCGTCTTTGGTC |  |  |
| CY-REOV_L1_2430R | TGACAGCACCCAATCGTAGT |  |  |
| CY-REOV_L1_3181F | GCTTGATGGTGTTAGCCTGT |  |  |
| CY-REOV_L1_3404R | GAGACATTTGTACCGGCGTC |  |  |
| CY-REOV_L1_356F | ACACCATTGTCGCTTCCTTC |  |  |
| CY-REOV_L1_3624F | CCAATACATTGATTTCGAAT |  |  |
| CY-REOV_L1_3878R | GATGAATATATATCACCCTT |  |  |
| CY-REOV_L1_586R | TAGATAACATCAGCGTAGGGAGTT |  |  |
| CY-REOV_L2_1363F | TCATGCGCTCACAATACGTT | L2:3820 | KM382260 |
| CY-REOV_L2_1494R | ACGTCAGCAGTCTGAGCCGC |  |  |
| CY-REOV_L2_1F | CTTTACTGATCCTACCATGCATGT |  |  |
| CY-REOV_L2_2375F | GAATGGTTGTCGCATACCCA |  |  |
| CY-REOV_L2_2514R | CTCAACCATCGCCTCCAATG |  |  |
| CY-REOV_L2_2872F | AGATCTAACCGTCGCGATGA |  |  |
| CY-REOV_L2_3340F | CACCGTACTGTCTCTGGGTT |  |  |
| CY-REOV_L2_357F | CTTAACCCCATCATCGCTGC |  |  |
| CY-REOV_L2_3814R | GGATGAATATGATCCACACC |  |  |
| CY-REOV_L2_574R | CGGACAGACGTCTCAGGACATAGA |  |  |
| CY-REOV_L3_1312F | CAGCTGACTTCGTTAATCAG | L3: 3944 | KM382261 |
| CY-REOV_L3_1416R | CATCCACAACTCACGAACGG |  |  |
| CY-REOV_L3_1F | GCTTTATTCCCCTAAGCGCGTTCC |  |  |
| CY-REOV_L3_2260F | TCCAATTCAGAACAGGCCGA |  |  |
| CY-REOV_L3_2444R | GCTGGGGTGCATGTCTTAAT |  |  |
| CY-REOV_L3_315F | ACCTGTCTCTTCGGAAGCTT |  |  |
| CY-REOV_L3_3217F | CAATCCCCTGGCTCCTCCTC |  |  |
| CY-REOV_L3_3388R | CGATATTCAACATCATGACT |  |  |
| CY-REOV_L3_3665F | AACGATGGTTCCCTGTTTGC |  |  |
| CY-REOV_L3_3936R | GATGAGTACTCCCGAGGGTG |  |  |
| CY-REOV_L3_534R | AATCCATTGTTTCGCGTTACCTTA |  |  |
| CY-REOV_M1_1064R | ACGATCCGAGAACAAGGTGA | M1:2277 | KM382262 |
| CY-REOV_M1_1187R | AGCCGACCATACTGCCAAGGCTT |  |  |
| CY-REOV_M1_1697F | ATTAGTCTTGAGTCGATGTT |  |  |
| CY-REOV_M1_1987R | CGCATGAATGAGAATGTTACATGT |  |  |
| CY-REOV_M1_1F | GCTTTAAATCGACATGGCGTACTT |  |  |
| CY-REOV_M1_2272R | GATGAATAATCTACTRCTACTAAT |  |  |
| CY-REOV_M1_509R | TGCCTTGTGAAATGTCGAAG |  |  |
| CY-REOV_M1_854F | CGTCAGATCAATACCTCCCTCAT |  |  |
| CY-REOV_M2_1048R | GTGTAAGCAACATCGAAACCAGTC | M2:2134 | KM382263 |
| CY-REOV_M2_1259F | TGGACAGTTGCTTCCCAGAT |  |  |
| CY-REOV_M2_1722F | TACAGGGTATCTTGGCGTTA |  |  |
| CY-REOV_M2_1F | GCTTATTTTGTGACAGGTCGCACT |  |  |
| CY-REOV_M2_2145R | GATGAATAAGCGGACAGGCCCGAC |  |  |
| CY-REOV_M2_584R | ACACTCTTTTCCCAGGGACC |  |  |
| CY-REOV_M2_915F | CGTCAGTGGATCTGATTTGTTCCA |  |  |
| CY-REOV_M3_1029R | CATCCATCAACATAGCACATTCAG | M3: 1983 | KM382264 |
| CY-REOV_M3_1205F | CAGCTGTTGATCGCTGTGGC |  |  |
| CY-REOV_M3_1302F | GCTCAAGGCTAAGCTTTCGG |  |  |
| CY-REOV_M3_1426R | GAGCCGGAACGTTCAAAATCTGAT |  |  |
| CY-REOV_M3_1569F | CGGAAAGGGAGATGAATCGC |  |  |
| CY-REOV_M3_1949R | GATGAATAGGGAYCCCCCAARGGG |  |  |
| CY-REOV_M3_1F | GCTTATTTTTCCACTAGCGTGCTT |  |  |
| CY-REOV_M3_591F | CGGTGTCGCTGACCTTTCGACTGA |  |  |
| CY-REOV_M3_598R | GTGTCAGTCGAAAGGTCAGC |  |  |
| CY-REOV_S1_1136F | TTGTAGTGATAACGCGGGGT | S1:1596 | KM382265 |
| CY-REOV_S1_1371R | GAACGCCGGTTGAGCCAGCAACAC |  |  |
| CY-REOV_S1_1577R | GATGAATAGCTGTCCTCGACGGCG |  |  |
| CY-REOV_S1_1F | GCTTTAATTTTGTTCTCGAGTGTC |  |  |
| CY-REOV_S1_442F | GCCGCATCTTGTAGCTGATC |  |  |
| CY-REOV_S1_577F | AGATCTCATGTCATCATTCACTA |  |  |
| CY-REOV_S1_648R | TTATGCTCTGGGTCATCGTCATGA |  |  |
| CY-REOV_S2_1303R | GATGATTARACCACGGCTGA | S2:1323 | KM382266 |
| CY-REOV_S2_1F | GCTTTAARAACCACGATGGC |  |  |
| CY-REOV_S2_314F | CTTGTCAGGTCTGGTTGCTG |  |  |
| CY-REOV_S2_421F | TAGAGAACTTGAAACCAAGTATCC |  |  |
| CY-REOV_S2_554R | CTCCCTGAAATCCTTACCATAACA |  |  |
| CY-REOV_S2_878F | CTCAGCTAAGCGCTTTGATA |  |  |
| CY-REOV_S3_1169R | GATGAATAGCTCCTGTCGATGCTC | S3:1180 | KM382267 |
| CY-REOV_S3_1F | GCTTAATTTTTCCTGTTGAGACGT |  |  |
| CY-REOV_S3_347F | CTCGAAGCTGCAAAACTGGT |  |  |
| CY-REOV_S3_525R | GAACAAGCTCAGCAGCGAGGAGAT |  |  |
| CY-REOV_S3_813F | AGTTGGAGTTGGACGCATTG |  |  |
| CY-REOV_S4_1165R | GATGAATACCATGTCTCGGC | S4:1184 | KM382268 |
| CY-REOV_S4_1F | GCTTATTTTGTCTTGGCGCGCAAG |  |  |
| CY-REOV_S4_290F | GAAGGTTGTTCGTGTGGGAC |  |  |
| CY-REOV_S4_518R | ACCTCAGACAGCTTATCCATAGAG |  |  |
| CY-REOV_S4_784F | TCACTCCGATTATGGCAGGT |  |  |
